# Preventive Effect of Pretreatment with Pitavastatin on Contrast-Induced Nephropathy in Patients with Renal Dysfunction Undergoing Coronary Procedure: PRINCIPLE-II Randomized Clinical Trial

**DOI:** 10.3390/jcm9113689

**Published:** 2020-11-17

**Authors:** Woong Chol Kang, Minsu Kim, Sang Min Park, Byeong-Keuk Kim, Byoung-Kwon Lee, Hyuck Moon Kwon

**Affiliations:** 1 Department of Cardiology, Gil Medical Center, Gachon University College of Medicine, Incheon 21565, Korea; kangwch@gilhospital.com; 2 Department of Cardiology, Chuncheon Sacred Heart Hospital, Hallym University College of Medicine, Chuncheon 24253, Korea; mskimgene@gmail.com; 3 Department of Cardiology, Nowon Eulji Medical Center, Eulji University College of Medicine, Seoul 01830, Korea; samipark@hanmail.net; 4 Department of Cardiology, Severance Cardiovascular Hospital, Yonsei University College of Medicine, Seoul 03722, Korea; kimbk@yuhs.ac; 5 Department of Cardiology, Gangnam Severance Hospital, Yonsei University College of Medicine, Seoul 06273, Korea; cardiobk@yuhs.ac

**Keywords:** pitavastatin, contrast-induced nephropathy, chronic kidney disease

## Abstract

This study aimed to evaluate the efficacy of pitavastatin pretreatment on contrast-induced nephropathy (CIN) in patients with chronic kidney disease (CKD) after a coronary procedure. This was a prospective, randomized, double-blinded, placebo-controlled, multicenter clinical trial. All consecutive 70 patients with CKD (eGFR < 60 mL/min/1.73 m^2^) were enrolled and randomized into two groups. Group I consisted of patients who were treated with statins (pitavastatin 4 mg/day) for seven days before and three days after the procedure (*n* = 37, 52.9%), and group II consisted of patients who were treated with a placebo (*n* = 33, 47.1%). The primary endpoint was the incidence of CIN, and the secondary endpoints were the change in serum creatinine (∆sCr) level and estimated glomerular filtration rate (∆eGFR) after the procedure. The mean age of the patients (males, 74%) was 70.4 ± 9.0 years. After the coronary procedure, the incidence of CIN was lower in group I than in group II, but the difference was not significant (5.4% vs. 9.1%, *p* = 0.661). The maximal ∆sCr was lower and the maximal ∆eGFR was higher in group I than in group II, but the difference was not significant (−0.11 ± 0.53 mg/dL and −0.04 ± 0.33 mg/dL, *p* = 0.678; 4.3 ± 11.2 mL/min/1.73 m^2^ and −2.9 ± 20.4 mL/min/1.73 m^2^, *p* = 0.161, respectively). This study showed the possibility of a clinical benefit of pretreatment with a high dose of pitavastatin for the prevention of CIN in patients with CKD after coronary procedure (ClinicalTrials.gov Identifier: NCT01871792).

## 1. Introduction

Contrast-induced nephropathy (CIN) is a major complication associated with adverse outcomes after coronary procedure in patients with chronic kidney disease (CKD) [1,2,3]. The mechanisms of CIN are not clearly defined. CIN may be related to renal tubular toxicity, vasoconstriction, and high oxidative stress [4]. Statins have favorable effects on endothelial function, nitric oxide production, and oxidative stress, which may be related to the development of CIN [5,6]. Hence, statins have been proposed for the prevention of CIN, given their antioxidant and anti-inflammatory properties [7,8]. However, their efficacy for the prevention of CIN is controversial [9,10,11,12,13]. The results of statin treatment might be influenced by the patient population, type or dose of statins, and duration of statin treatment before and after coronary procedure. This study aimed to evaluate the efficacy of the administration of short-term high-dose pitavastatin, added to routine intravenous hydration, in reducing the occurrence of CIN in patients with CKD undergoing a planned coronary procedure.

## 2. Materials and Methods

**Figure 1:**
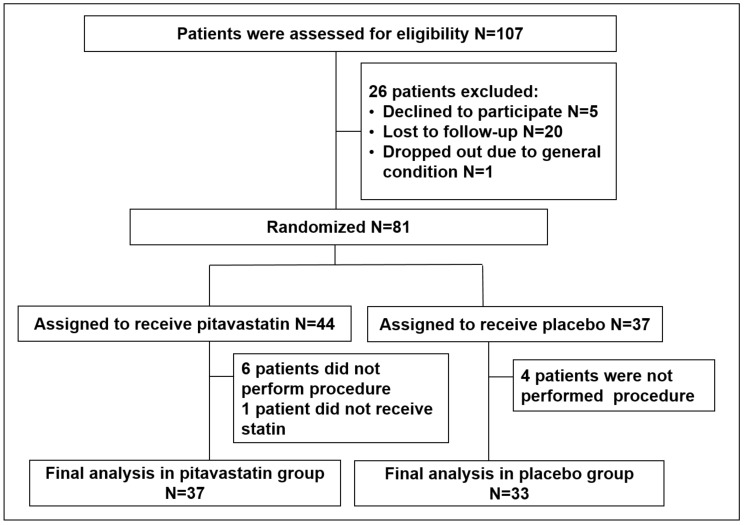
Patient flowchart.

This was a prospective, randomized, double-blinded, placebo-controlled, multicenter clinical trial performed in patients with CKD undergoing elective coronary angiography and/or intervention (NCT01871792). From September 2013 to February 2016, 107 patients who had an estimated glomerular filtration rate (eGFR) of <60 mL/min underwent planned coronary procedures and were considered eligible for inclusion in our study. Patients undergoing current treatment with a statin, those with contraindication to statin treatment, those administered contrast media (within 10 days of entry into the study), those with end-stage renal failure requiring dialysis, and those who refused to provide informed consent were excluded. Of 107 patients, 26 patients were excluded due to declining to participate, loss of follow-up and poor general condition. Finally, 81 patients were enrolled in the study (Figure 1).

Randomization was performed using computerized assignment in a consecutive manner. In total, 44 patients were randomized to receive 4 mg pitavastatin (Livalo, Choongwae Pharma, Seoul, Republic of Korea) every morning, from 7 days before to 3 days after coronary procedure (total dose of 40 mg of pitavastatin over 10 days), and 37 patients were randomized to receive a matching placebo for the same period before and after coronary procedure. Statin therapy was resumed in both groups 3 days after the procedure, following completion of the study endpoints. Standard hydration therapy was administered at the discretion of the physician; the therapy included isotonic saline solution (0.9% sodium chloride, 1 mL/kg/h), and it was started 12 h before and continued for 24 h after the procedure. The iso-osmolar, nonionic contrast medium iodixanol (320 mg iodine/mL; Visipaque, GE Healthcare, Piscataway, NJ, USA) was administered during all procedures. Blood samples were collected to measure parameters before randomization and at 24 h and 48 h after the procedure. Renal function was measured using eGFR in all patients. The Institutional Review Board of Gachon University Gil Medical Center approved this study (GDIRB 2013-21), and all patients provided written informed consent prior to enrollment.

Serum creatinine (sCr) concentration was assessed at the time of hospital admission and at 24 and 48 h after the coronary procedure. The primary endpoint was the development of CIN, defined as ≥0.5 mg/dL or a ≥25% increase in sCr concentration above baseline within 48 h after exposure to the contrast medium. The secondary endpoints were the maximal change in sCr and eGFR after exposure to the contrast medium. Clinical outcomes included the following events occurring within 30 days of the procedure: (1) all-cause death, and (2) dialysis or hemofiltration due to symptoms or signs of uremic syndrome or management of refractory hypervolemia, hyperkalemia, or acidosis. All patients underwent a follow-up evaluation at the clinic or via telephonic contact at 30 days.

Statistical analysis was performed using SPSS 20.0 (SPSS, Inc., Chicago, IL, USA). Categorical variables were presented as counts and percentages and compared using Chi-square or Fisher’s exact test. Continuous variables were compared using the Student’s *t*-test for normally distributed values; the Mann–Whitney U-test was used for other variables. All *p*-values were two tailed, and statistical significance was defined as a *p*-value < 0.05.

## 3. Results

### 3.1. Baseline Characteristics between Patients Pretreated with Pitavastatin and Placebo

The mean age of the patients (74% male) was 70.4 ± 9.0 years. Their mean baseline sCr and eGFR were 1.59 ± 0.75 mg/dL and 47.5 ± 20.4 mL/min/1.73 m^2^, respectively. Of these, 15.7% of patients had severe renal impairment, with eGFR < 30 mL/min/1.73 m^2^. The baseline clinical, laboratory, and procedural characteristics did not significantly differ between the groups (Table 1). The proportion of patients with diabetes mellitus, dyslipidemia, previous percutaneous coronary intervention (PCI), and history of cerebrovascular accident was higher in group I than in group II, however the difference was not significant. The values of left ventricular ejection fraction and lipid profile were also similar between the groups. PCI was performed in 40.5% of patients in group I and 39.4% of patients in group II. The mean sCr and eGFR values are listed in Table 2. The baseline sCr (1.68 ± 1.03 mg/dL vs. 1.53 ± 0.62 mg/dL, *p* = 0.575) and eGFR (46.5 ± 15.1 mL/min/1.73 m^2^ vs. 49.1 ± 23.9 mL/min/1.73 m^2^, *p* = 0.677) levels were similar between the groups (Table 2).

### 3.2. Preventive Effect of Statins on CIN and Clinical Outcomes during Follow-Up

**Figure 2:**
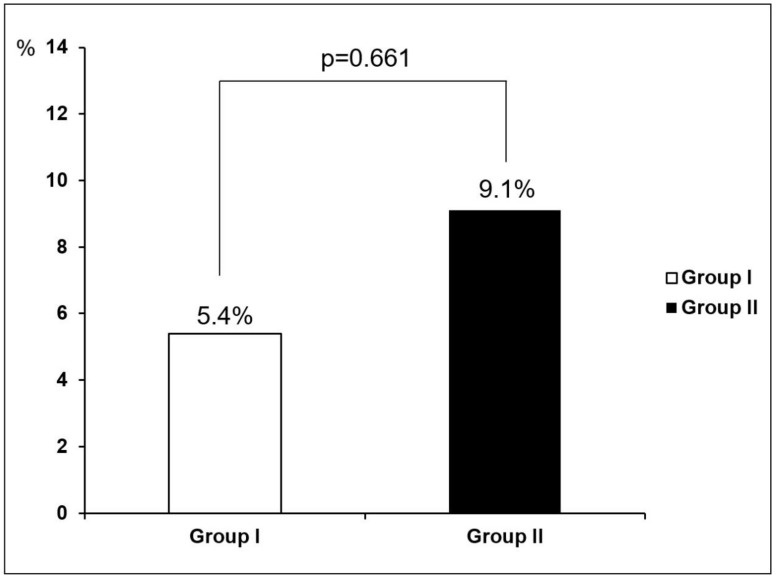
Primary endpoint: CIN occurrence.

The primary CIN endpoint occurred in a total of five patients (7.1%): two patients (5.4%) in group I and three patients (9.1%) in group II (*p* = 0.661; Figure 2). The changes in sCr and eGFR values after the coronary procedure are listed in Table 2. The maximal change in sCr was lower and maximal change in eGFR was higher in group I than in group II, but the difference was not significant (−0.11 ± 0.53 mg/dL and −0.04 ± 0.33 mg/dL, *p* = 0.678; 4.3 ± 11.2 mL/min/1.73 m^2^ and −2.9 ± 20.4 mL/min/1.73 m^2^, *p* = 0.161, respectively). No clinical event, including death, dialysis, or hemofiltration due to symptoms or signs of uremic syndrome or management of refractory hypervolemia, hyperkalemia, or acidosis, was observed after the procedure and during the 30-day follow-up period.

## 4. Discussion

To the best of our knowledge, this is the first prospective, randomized, double-blinded, placebo-controlled clinical trial to evaluate the efficacy of pitavastatin therapy for the prevention of CIN in patients with mild-to-moderate CKD. In this trial, we observed that periprocedural administration of pitavastatin 4 mg daily before and after a coronary procedure (10 days) might reduce the incidence of CIN in patients with CKD. The maximal change in sCr was lower and maximal change in eGFR was higher in patients pretreated with pitavastatin after coronary procedure. These findings suggest that pretreatment with pitavastatin prevents worsening of renal function in patients with CKD after use of contrast medium. Because CIN is a severe complication in patients with already impaired kidney function, such as in patients included in our study, these results have clinical significance [1,2,3].

**Figure 3:**
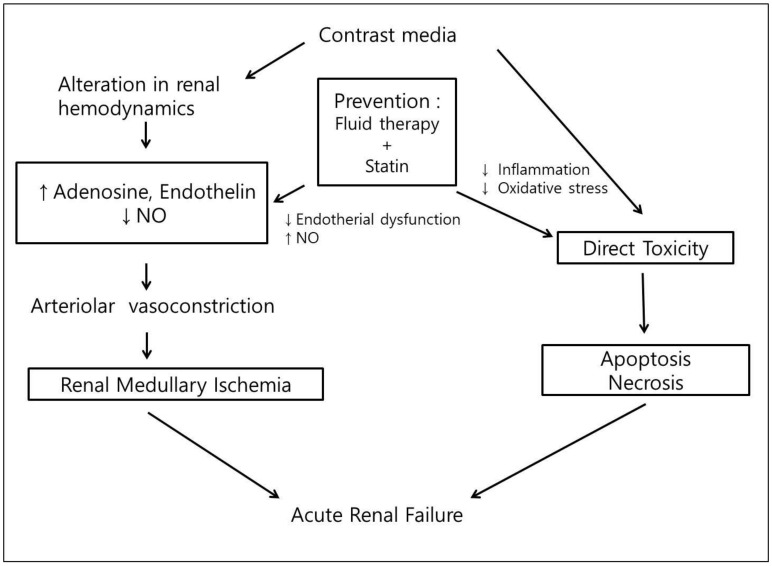
Potential mechanisms of beneficial effects of statins on the CIN. NO: nitric oxide.

Although the mechanisms of CIN are not clearly defined, endothelin-mediated intensive vasoconstriction, nitric oxide-mediated inhibition of vasodilation, and oxidative stress caused by reactive oxygen species are responsible for the development of CIN [4]. Statins are principal therapeutic agents in lowering blood cholesterol. The beneficial and protective effects on endothelial cells may lead to increased synthesis of nitric oxide, regression of atherosclerotic plaque, and inhibition of inflammatory process in ischemic heart disease and renal dysfunction [14,15,16]. Much evidence has suggested that statins may play a crucial role in the prevention of CIN through their pleiotropic effect. [7,8] (Figure 3).

However, previous studies regarding the impact of pretreatment with statins on the incidence of CIN have showed conflicting results [9,10,11,12,13]. The prevention of CIN using short-term high-dose simvastatin in patients with CKD undergoing coronary angiography study was the first randomized, prospective trial focused on the role of short-term statin therapy for the prevention of CIN [10]. However, the results of this study showed that short-term pretreatment (48 h) with high-dose simvastatin (40 mg twice a day) did not prevent the deterioration of renal function in patients with CKD who received intravenous hydration. In another study, short-term administration (48 h before and 48 h after contrast exposure) of high doses of atorvastatin (80 mg/day), in addition to standard intravenous hydration and oral N-acetylcysteine, also did not reduce the occurrence of CIN in patients with CKD [12]. On the contrary, Han et al. reported that rosuvastatin (10 mg/day, for 5 days: 2 days before and 3 days after the procedure) significantly reduced the risk of CIN in patients with type 2 diabetes mellitus and concomitant CKD who were undergoing coronary/peripheral arterial angiography with or without percutaneous intervention [9]. Liang et al. also reported, in meta-analysis data including 15 randomized controlled trials, that moderate- or high-dose rosuvastatin treatment could reduce the incidence of CIN in patients undergoing coronary angiography or PCI [11]. Another meta-analysis by Yang et al. compared rosuvastatin treatment with no-statin treatment in preventing CIN, and found that compared to the control group, patients treated with rosuvastatin had a 51% lower risk of CIN (OR = 0.49, 95% CI = 0.37–0.66, *p* < 0.001) [13]. However, this meta-analysis showed that rosuvastatin treatment had no effect in terms of preventing CIN in patients with CKD undergoing elective coronary procedure (OR = 0.81, 95% CI = 0.41–1.61, *p* = 0.55). These conflicting results might be attributed to the differences in the definitions of CIN, study design, patient population, statins used, and the dosage and duration of statin pretreatment in the different studies.

The pleiotropic effects of different statins vary. Whether rosuvastatin is superior to atorvastatin in the prevention of CIN is not clear. A large prospective, observational study by Liu et al. compared the effects of rosuvastatin and atorvastatin for the prevention of CIN in patients with CKD undergoing PCI (273 patients received rosuvastatin 10 mg and 805 patients received atorvastatin 20 mg), and demonstrated that the preventive effect of both statins for the occurrence of CIN was similar [17]. In consideration of the complex mechanisms of CIN, rosuvastatin and atorvastatin have their own characteristics in the prevention of CIN.

Pitavastatin was no less effective than atorvastatin and simvastatin in presumed equipotent dosages, and was superior to pravastatin, in lowering LDL-C levels [18,19]. Pitavastatin provided sustained LDL-C-lowering efficacy and was associated with short- and longer-term improvements in several other lipid parameters [20,21]. Short- and longer-term outcomes in studies on Asian patients were consistent with these findings [22,23]. Pitavastatin was also generally well tolerated and did not appear to adversely affect glucose metabolism parameters. Thus, pitavastatin is an effective treatment option in adults with primary hypercholesterolemia and combined dyslipidemia, including those at risk of developing type 2 diabetes. However, there are no data evaluating the efficacy and safety of pitavastatin on the prevention of CIN in patients with CKD undergoing a coronary procedure. The current study is of value through its double blinded, placebo-controlled design, enrolling two groups of patients with a relatively homogenous risk profile. Furthermore, to the best of our knowledge, this is the first study evaluating the role of pitavastatin for the prevention of CIN in patients with mild-to-moderate CKD undergoing elective coronary procedure. Moreover, in this study, high-dose pitavastatin was administered after coronary procedure for a relatively long period (4 mg/day for 10 days), compared with previous studies. Our results show that pitavastatin might reduce the risk of CIN and preserve renal function, even in patients with normal lipid levels. Therefore, the present study indicated that in addition to hydration, pitavastatin should be administered to all patients with mild-to-moderate CKD who receive contrast medium.

Our study has a number of limitations. Due to difficulty of enrollment, the number of patients in our study was relatively small. Therefore, our study does not have statistical power to prove our hypothesis. Due to the heterogeneity of patients to develop CIN, further large-scale studies are required to explore the benefits of pitavastatin pretreatment for prevention of CIN according to baseline eGFR. Although pitavastatin was administered for 10 days in the periprocedural period, which is longer than the period of administration in previous studies, the duration of statin treatment may be insufficient for exercising a protective effect against acute renal injury. In addition, the accurate dose of pitavastatin is another issue, because most previous studies showing the protective effect of statin for the prevention of CIN used high doses of the most potent statins, such as atorvastatin or rosuvastatin.

## 5. Conclusions

This study showed a possibility of clinical benefit of pretreatment with high-dose pitavastatin for prevention of CIN in patients with CKD after a coronary procedure.

## Figures and Tables

**Table 1:** Baseline characteristics of patients.

	Group I (*n* = 37)	Group II (*n* = 33)	*p*-Value
Age (years)	70.8 ± 8.9	70.0 ± 9.2	0.720
Height (cm)	162.3 ± 10.3	163.1 ± 7.2	0.702
Weight (kg)	66.5 ± 11.2	65.1 ± 9.9	0.575
BMI (kg/m^2^)	25.2 ± 3.3	24.4 ± 2.7	0.265
Male, *n* (%)	26 (70.3)	26 (78.8)	0.585
Smoker, *n* (%)	10 (27.0)	7 (21.2)	0.592
Hypertension, *n* (%)	28 (75.7)	22 (66.7)	0.438
Diabetes mellitus, *n* (%)	23 (62.2)	16 (48.5)	0.336
Dyslipidemia, *n* (%)	11 (29.7)	5 (15.2)	0.167
Previous MI, *n* (%)	7 (18.9)	5 (15.2)	0.758
Previous PCI, *n* (%)	15 (40.5)	8 (24.2)	0.204
Previous CVA, *n* (%)	10 (27.0)	4 (12.1)	0.144
Congestive heart failure, *n* (%)	7 (23.3)	6 (25.0)	1.000
Myocardial ischemia, *n* (%)			0.728
Stable angina	23 (62.2)	21 (63.6)	
Unstable angina	6 (16.2)	7 (21.2)	
NSTEMI	8 (21.6)	5 (15.2)	
LVEF, %	58.1 ± 10.1	55.6 ± 9.3	0.342
Medications, *n* (%)			
Aspirin	37 (100)	33 (100)	1.000
Clopidogrel	25 (67.6)	21 (63.6)	0.651
ACEI or ARB	23 (62.2)	18 (54.5)	0.628
Beta-blocker	17 (45.9)	16 (48.5)	0.687
CCB	12 (32.4)	5 (15.2)	0.105
Laboratory determinations, mg/dL			
Total cholesterol	162.9 ± 36.6	178.2 ± 35.1	0.117
Triglyceride	105.1 ± 97.4	108.7 ± 134.5	0.898
HDL-C	30.3 ± 23.2	35.5 ± 28.4	0.417
LDL-C	85.6 ± 35.3	93.2 ± 35.9	0.485
HgA1C	6.6 ± 1.0	6.5 ± 1.3	0.826
Fasting glucose	115.6 ± 65.5	104.9 ± 77.0	0.536
Procedure, *n* (%)			1.000
CAG	22 (59.5)	20 (60.6)	
PCI	15 (40.5)	13 (39.4)	

BMI: body mass index; MI: myocardial infarction; PCI: percutaneous coronary intervention; CVA: cerebrovascular accident; NSTEMI: non-ST elevation myocardial infarction; LVEF: left ventricular ejection fraction; ACEI: angiotensin-converting enzyme inhibitor; ARB: angiotensin II receptor blocker; CCB: calcium channel blocker; HDL-C: high-density lipoprotein cholesterol; LDL-C: low-density lipoprotein cholesterol; HgA1C: Hemoglobin A1C; CAG: coronary angiography.

**Table 2:** Biochemical characteristics of patients before and after coronary procedure.

	Group I (*n* = 37)	Group II (*n* = 33)	*p*-Value
Baseline			
sCr, mg/dL	1.68 ± 1.03	1.53 ± 0.62	0.575
eGFR, mL/min/1.73 m^2^	46.5 ± 15.1	49.1 ± 23.9	0.677
Follow-up			
24 h sCr, mg/dL	1.62 ± 0.88	1.40 ± 0.67	0.414
24 h ∆sCr, mg/dL	−0.13 ± 0.39	−0.10 ± 0.24	0.784
48 h sCr, mg/dL	1.49 ± 0.66	1.38 ± 0.53	0.573
48 h ∆sCr, mg/dL	−0.08 ± 0.44	−0.06 ± 0.23	0.870
Maximal ∆sCr, mg/dL	−0.11±0.53	−0.04±0.33	0.678
24 h eGFR, mg/dL, mL/min/1.73 m^2^	53.3 ± 17.9	48.5 ± 17.3	0.433
24 h ∆eGFR, mL/min/1.73 m^2^	5.7 ± 12.1	−1.9 ± 22.3	0.205
48 h eGFR, mg/dL, mL/min/1.73 m^2^	52.0 ± 16.6	50.1 ± 17.8	0.753
48 h ∆eGFR, mL/min/1.73 m^2^	4.3 ± 10.1	−1.5 ± 24.9	0.371
Maximal ∆eGFR-all, mL/min/1.73 m^2^	4.3 ± 11.2	−2.9 ± 20.4	0.161
Maximal ∆eGFR, mL/min/1.73 m^2^	1.9 ± 11.0	−8.2 ± 28.5	0.212

sCr: serum creatinine; eGFR: estimated glomerular filtration rate; ∆sCr: change in serum creatinine level; ∆eGFR: change in estimated glomerular filtration rate; CIN: contrast-induced nephropathy.

## References

[1] McCullough P.A., Adam A., Becker C.R., Davidson C., Lameire N., Stacul F., Tumlin J., CIN Consensus Working Panel (2006). Epidemiology and prognostic implications of contrast-induced nephropathy. Am. J. Cardiol..

[2] Mehran R., Aymong E.D., Nikolsky E., Lasic Z., Iakovou I., Fahy M., Mintz G.S., Lansky A.J., Moses J.W., Stone G.W. (2004). A simple risk score for prediction of contrast-induced nephropathy after percutaneous coronary intervention: Development and initial validation. J. Am. Coll. Cardiol..

[3] Seeliger E., Sendeski M., Rihal C.S., Persson P.B. (2012). Contrast-induced kidney injury: Mechanisms, risk factors, and prevention. Eur. Heart J..

[4] John S., Schneider M.P., Delles C., Jacobi J., Schmieder R.E. (2005). Lipid-independent effects of statins on endothelial function and bioavailability of nitric oxide in hypercholesterolemic patients. Am. Heart J..

[5] Persson P.B., Hansell P., Liss P. (2005). Pathophysiology of contrast medium-induced nephropathy. Kidney Int..

[6] Tumlin J., Stacul F., Adam A., Becker C.R., Davidson C., Lameire N., McCullough P.A., CIN Consensus Working Panel (2006). Pathophysiology of contrast-induced nephropathy. Am. J. Cardiol..

[7] Attallah N., Yassine L., Musial J., Yee J., Fisher K. (2004). The potential role of statins in contrast nephropathy. Clin. Nephrol..

[8] Ridker P.M., Rifai N., Clearfield M., Downs J.R., Weis S.E., Miles J.S., Gotto A.M., Air Force/Texas Coronary Atherosclerosis Prevention Study Investigators (2001). Measurement of C-reactive protein for the targeting of statin therapy in the primary prevention of acute coronary events. N. Engl. J. Med..

[9] Han Y., Zhu G., Han L., Hou F., Huang W., Liu H., Gan J., Jiang T., Li X., Wang W. (2014). Short-term rosuvastatin therapy for prevention of contrast-induced acute kidney injury in patients with diabetes and chronic kidney disease. J. Am. Coll. Cardiol..

[10] Jo S.H., Koo B.K., Park J.S., Kang H.J., Cho Y.S., Kim Y.J., Youn T.J., Chung W.Y., Chae I.H., Choi D.J. (2008). Prevention of radiocontrast medium-induced nephropathy using short-term high-dose simvastatin in patients with renal insufficiency undergoing coronary angiography (PROMISS) trial—A randomized controlled study. Am. Heart J..

[11] Liang M., Yang S., Fu N. (2017). Efficacy of short-term moderate or high-dose rosuvastatin in preventing contrast-induced nephropathy: A meta-analysis of 15 randomized controlled trials. Medicine.

[12] Toso A., Maioli M., Leoncini M., Gallopin M., Tedeschi D., Micheletti C., Manzone C., Amato M., Bellandi F. (2010). Usefulness of atorvastatin (80 mg) in prevention of contrast-induced nephropathy in patients with chronic renal disease. Am. J. Cardiol..

[13] Yang Y., Wu Y.X., Hu Y.Z. (2015). Rosuvastatin Treatment for Preventing Contrast-Induced Acute Kidney Injury after Cardiac Catheterization: A Meta-Analysis of Randomized Controlled Trials. Medicine.

[14] Katsiki N., Mikhailidis D.P., Banach M. (2019). Lipid-lowering agents for concurrent cardiovascular and chronic kidney disease. Expert Opin. Pharmacother..

[15] Severino P., D’Amato A., Pucci M., Infusino F., Adamo F., Birtolo L.I., Netti L., Montefusco G., Chimenti C., Lavalle C. (2020). Ischemic Heart Disease Pathophysiology Paradigms Overview: From Plaque Activation to Microvascular Dysfunction. Int. J. Mol. Sci..

[16] Severino P., D’Amato A., Pucci M., Infusino F., Birtolo L.I., Mariani M.V., Lavalle C., Maestrini V., Mancone M., Fedele F. (2020). Ischemic Heart Disease and Heart Failure: Role of Coronary Ion Channels. Int. J. Mol. Sci..

[17] Liu Y., Liu Y.H., Tan N., Chen J.Y., Zhou Y.L., Li L.W., Duan C.Y., Chen P.Y., Luo J.F., Li H.L. (2014). Comparison of the efficacy of rosuvastatin versus atorvastatin in preventing contrast induced nephropathy in patient with chronic kidney disease undergoing percutaneous coronary intervention. PLoS ONE.

[18] Liu P.Y., Lin L.Y., Lin H.J., Hsia C.H., Hung Y.R., Yeh H.I., Wu T.C., Chen J.Y., Chien K.L., Chen J.W. (2013). Pitavastatin and Atorvastatin double-blind randomized comPArative study among hiGh-risk patients, including thOse with Type 2 diabetes mellitus, in Taiwan (PAPAGO-T Study). PLoS ONE.

[19] Ose L., Budinski D., Hounslow N., Arneson V. (2009). Comparison of pitavastatin with simvastatin in primary hypercholesterolaemia or combined dyslipidaemia. Curr. Med. Res. Opin..

[20] Eriksson M., Budinski D., Hounslow N. (2011). Long-term efficacy of pitavastatin versus simvastatin. Adv. Ther..

[21] Sponseller C.A., Morgan R.E., Kryzhanovski V.A., Campbell S.E., Davidson M.H. (2014). Comparison of the lipid-lowering effects of pitavastatin 4 mg versus pravastatin 40 mg in adults with primary hyperlipidemia or mixed (combined) dyslipidemia: A Phase IV, prospective, US, multicenter, randomized, double-blind, superiority trial. Clin. Ther..

[22] Teramoto T. (2011). Pitavastatin: Clinical effects from the LIVES Study. Atheroscler. Suppl..

[23] Yokote K., Shimano H., Urashima M., Teramoto T. (2011). Efficacy and safety of pitavastatin in Japanese patients with hypercholesterolemia: LIVES study and subanalysis. Expert Rev. Cardiovasc. Ther..

